# PD202 Reimbursement Success For Pharmaceutical Products With Noncomparative Data: Are Health Technology Assessment Bodies Well Suited To Embrace Innovations?

**DOI:** 10.1017/S0266462324004240

**Published:** 2025-01-07

**Authors:** Talya Kolukfaki, Reut Fineberg, Katherine Leong, Richard Macaulay

## Abstract

**Introduction:**

Randomized controlled trials are ideal for securing marketing authorization (MA) for pharmaceuticals. However, ethical and feasibility constraints have led to the use of noncomparative trials by regulatory bodies to balance evidence uncertainty and patient access, especially for medicines addressing rare diseases or unmet healthcare needs. This research focused on pharmaceuticals approved with noncomparative data and how these translate into reimbursement outcomes.

**Methods:**

Indications approved by the European Medicines Agency (EMA) between January 2018 and October 2023 based on noncomparative data were identified. Generics and biosimilars were excluded. Approvals based on non-comparative trial data were identified from European public assessment reports and from “Procedural steps taken and scientific information after authorization” documents. The latter were also used to identify changes in MAs after submission of new data. Missing trial information was extracted from ClinicalTrials.gov. Health technology assessment (HTA) outcomes from the EU4Health programme and the UK were extracted from agency websites.

**Results:**

The EMA approved 46 indications based on noncomparative data: 18 of the 46 (39%) received full MA, 23 (50%) received conditional MA (CMA), and five (11%) had a CMA converted to full MA with additional data. The results for HTAs conducted in various countries were as follows:

- England: 29 of 46 (63%) indications assessed, (23/29 [79%] recommended);
- Scotland: 28 of 46 (61%) indications assessed, (20/28 [71%] recommended);
- France: 33 of 46 (72%) indications assessed, (7/33 [21%] received Amélioration du Service Médical Rendu level I to III);
- Germany: 37 of 46 (80%) indications assessed, (2/37 [5%] achieved additional benefit);
- Italy: 37 of 46 (80%) products assessed, (33/37 [89%] reimbursed); and
- Spain: 34 of 46 (74%) products assessed, (23/34 [68%] reimbursed).

**Conclusions:**

Indications approved by the EMA with noncomparative data achieved mixed HTA outcomes in the EU4Health programme and the UK. More negative outcomes were seen in markets with clinical-benefit payer archetypes (France and Germany). All conversions from CMA to full MA occurred within the last 24 months.

